# Should Serum Protein Electrophoresis Be a Surrogate for Liver Biopsy in Some Cases of Alpha_1_ Antitrypsin Deficiency?

**DOI:** 10.1155/2017/2705131

**Published:** 2017-09-28

**Authors:** Newton Key Hokama, Marcelo Padovani de Toledo Moraes, Paula de Oliveira Montandon Hokama, Fernando Gomes Romeiro

**Affiliations:** ^1^Internal Medicine Department, Hematology Division, Botucatu Medical School, Universidade Estadual Paulista (UNESP), Botucatu, SP, Brazil; ^2^Pathology Department, Botucatu Medical School, Universidade Estadual Paulista (UNESP), Botucatu, SP, Brazil; ^3^Internal Medicine Department, Gastroenterology Division, Botucatu Medical School, Universidade Estadual Paulista (UNESP), Botucatu, SP, Brazil

## Abstract

Most patients with alpha_1_ antitrypsin deficiency do not receive this diagnosis until developing severe complications, in particular when respiratory symptoms are absent. This is a reason for making alpha_1_ antitrypsin deficiency a possible diagnosis among patients with cryptogenic cirrhosis or other conditions of liver disease without a clear etiology. In this report, a case of cryptogenic cirrhosis is presented, showing the role of serum protein electrophoresis in the diagnosis, which was made before liver biopsy. Therefore, the possibility of using a typical pattern of serum protein electrophoresis as a surrogate for liver biopsy in alpha_1_ antitrypsin deficiency is discussed.

## 1. Introduction

Alpha_1_ antitrypsin deficiency (AATD) was first diagnosed in 1963, when Carl-Bertil Laurell and Sten Eriksson noticed that the *α*1-protein band in serum protein electrophoresis was absent in five patients [1]. They pointed out two relevant facts: the use of serum protein electrophoresis to make the diagnosis and the role of case reports when assessing diseases that are usually underdiagnosed. Despite all the progress achieved after this initial description, the disease is still unrecognized until the occurrence of complications that bring the patients to specialists [2, 3].

The enzyme alpha_1_ antitrypsin is a serine protease inhibitor capable of neutralizing the proteolysis caused by the neutrophil elastase released from the azurophilic granules of these cells [2]. AATD has been included in the group of diseases caused by misfolding and intracellular polymerization of serpin superfamily members [1, 4]. Patients stricken by serpinopathies suffer one of two distinct pathophysiological patterns: loss-of-function deficiencies, such as what occurs in emphysema, or a toxic effect due to accumulation of abnormal proteins, such as in cirrhosis [2].

The disease is more common among Caucasian people, accounting for 1.1% of adult liver transplants in the UK [3], but it is uncommon in Asian countries [2]. The mutation found in patients with AATD leads to protein retention within hepatocytes, which in turn causes liver damage [4]. The lack of alpha_1_ antitrypsin is associated with panlobular basal emphysema, which is the primary cause that leads to the diagnosis and can be found in 1-2% of patients with chronic obstructive pulmonary disease (COPD) [4]. However, it may be difficult to stablish the diagnosis when the patients do not present with respiratory symptoms, because the protein retention into the liver is often asymptomatic. Therefore, in this report, we present a patient with AATD with a typical pattern observed in serum protein electrophoresis, which in our opinion could be a surrogate for liver biopsy in similar cases in which liver biopsy could be avoided.

## 2. Case Report

An asymptomatic 44-year-old woman with ascites and other portal hypertension findings in a recent ultrasonography came to medical consultation. Serological tests for viral hepatitis were negative and she was not an alcohol consumer.

**Figure 1:**
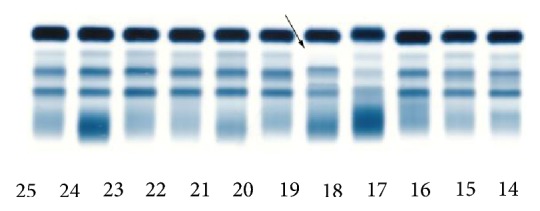
Serum protein electrophoresis investigation with reduced alpha_1_ globulin band (arrow, column 18).

Serum protein electrophoresis revealed reduced alpha_1_ globulin band (Figure 1).

**Figure 2:**
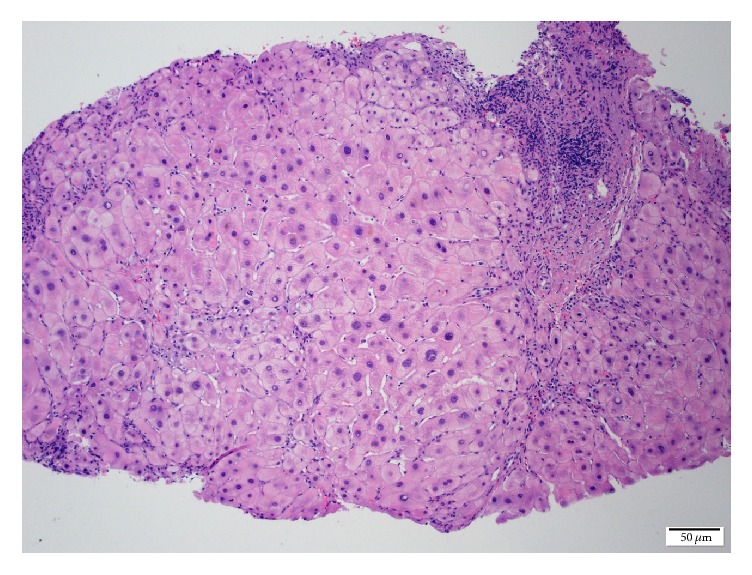
Liver biopsy showing nodules and fibrous septa associated with effacement of lobular architecture in Hematoxylin-Eosin staining.

**Figure 3:**
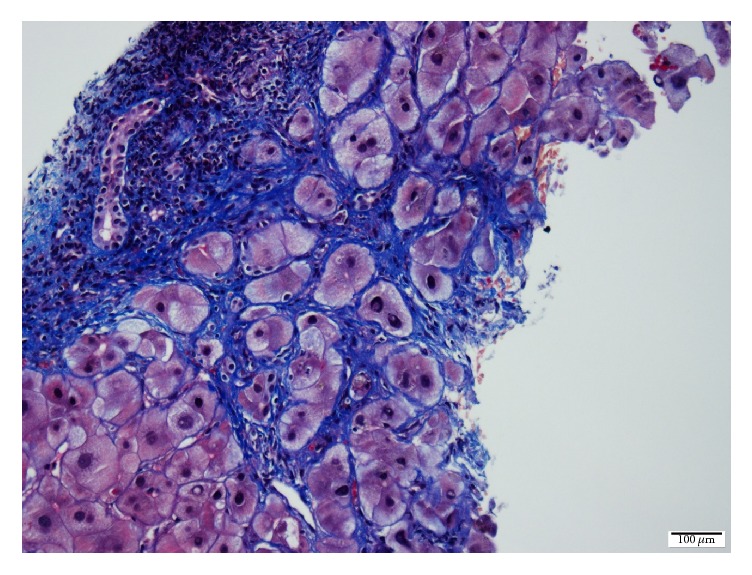
Liver biopsy showing nodules and fibrous septa associated with effacement of lobular architecture in Masson's trichrome stain.

**Figure 4:**
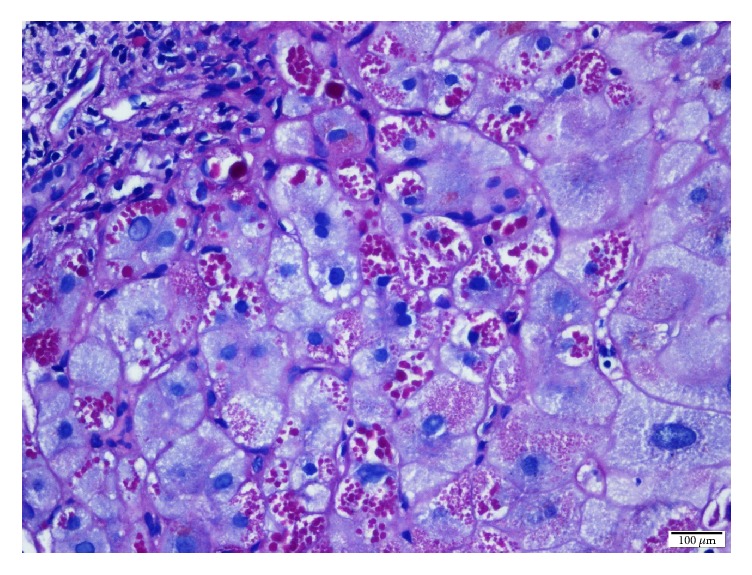
Liver biopsy showing the periportal localization of periodic acid-Schiff positive inclusion bodies, confirming the diagnosis of alpha_1_ antitrypsin deficiency.

Liver biopsy showed nodules and fibrous septa with effacement of lobular architecture in Hematoxylin-Eosin (Figure 2) and Masson's trichrome (Figure 3) stains. Periportal localization of periodic acid-Schiff positive inclusion bodies was observed (Figure 4), confirming the diagnosis of alpha_1_ antitrypsin deficiency. Her liver biopsy was uneventful, but before any additional exams such as genetic analysis, she passed away due to a septic shock.

## 3. Discussion

In spite of the success in establishing the diagnosis when patients with AATD are submitted to liver transplantation, only few cases receive this diagnosis [5–7]. Most physicians do not test patients for AATD, spending more than five years until the disease is recognized [2]. Since only 10% of patients or less are properly diagnosed, AATD is still an uncommon cause of liver disease submitted to liver transplantation [8]. After AATD suspicion, the diagnosis usually begins by testing the serum levels of alpha_1_ antitrypsin in patients with established risk factors, but sometimes the disease is only revealed after liver biopsy [9].

The associated findings are COPD, emphysema or asthma with incompletely reversible airflow obstruction, bronchiectasis or chronic liver disease without a clear etiology (such as cryptogenic cirrhosis), persistent airway obstruction, necrotizing panniculitis, c-ANCA-positive vasculitis, family history of AATD, COPD, or liver disease (which could be attributed to AATD), and people at high risk for AATD [2]. Other reasons could be considered from genetic tests suggesting a high risk for AATD.

Liver disease caused by AATD occurs in patients older than those who have a pulmonary disease, raising the hypothesis that when they do not develop emphysema in younger ages it could be a risk factor for liver disease in middle age [2]. In this setting, the absence of pulmonary findings can be a barrier to the diagnosis, leading to cryptogenic cirrhosis and liver cancer. Additionally, when patients have infections or any other inflammatory conditions, serum lab tests can be unreliable, because alpha_1_ antitrypsin levels can increase in the presence of inflammation [10]. Once the patient developed liver cirrhosis, some complications such as ascites, thrombocytopenia, and/or prothrombin time alterations make it difficult to obtain adequate liver samples through percutaneous liver biopsy, sometimes precluding the diagnosis of AATD. For these patients, serum protein electrophoresis, genetic tests, and transjugular liver biopsy could be performed in order to search for the initial liver disease.

Most cases of liver disease are associated with homozygosity for the Z mutant allele, also called PiZZ, in which the anomalous protein folds improperly inside hepatocytes. Consequently, approximately 85% of the molecules are retained into these cells instead of being secreted [11]. As a result, the low alpha_1_ antitrypsin serum level can be easily detected by serum protein electrophoresis [11].

Therefore, when a typical pattern is observed in serum protein electrophoresis, it can be a relevant clue to suggest the presence of AATD. However, it could never be enough to establish the diagnosis without further exams, such as genetic tests. Worth mentioning is that when the pattern observed is not so specific as shown in this report, the disease could be never ruled out, and genetic tests and/or liver biopsies should be carried out. As AATD is still a cause for high mortality rates associated with liver and lung complications, the proper diagnosis can save lots of patients stricken by this disease [12, 13].

In conclusion, we suggest that every patient with cryptogenic cirrhosis should be submitted to specific tests considering AATD, with special attention to serum protein electrophoresis. If a typical pattern is observed in this exam, the diagnosis of AATD must be suspected, leading to worthy indications of additional exams such as genetic tests, perhaps avoiding the need of invasive exams such as liver biopsy.
